# Acute Methotrexate Ingestions in Adults: A Report of Serious Clinical Effects and Treatments

**DOI:** 10.1155/2014/214574

**Published:** 2014-04-16

**Authors:** Vikhyat S. Bebarta, Matthew D. Hensley, Douglas J. Borys

**Affiliations:** ^1^Medical Toxicology, San Antonio Military Medical Center, San Antonio, TX 78234, USA; ^2^San Antonio Military Medical Center, San Antonio, TX 78234, USA; ^3^School of Pharmacy, Concordia University, Mequon, WI, USA

## Abstract

*Objective.* Limited reported data have reports effects after acute ingestion of methotrexate. Treatment recommendations do not differentiate between exposure routes. Our objective was to determine the frequency of significant toxicity effects and use of therapy after methotrexate ingestion in adults. *Methods.* We performed a retrospective study on adult cases reported to 6 poison centers over 6 years (2000–2005) which exceed 180,000 exposures/year. Variables collected included demographics, dosages ingested, coingestions, clinical effects, and therapies with outcomes. *Results.* Sixty-three patients examined over the 6-year period met inclusion criteria. No patient in the series received dialysis or died. The mean dose ingested for all patients was 24 mg (range 2.5–100 mg) and the mean dose for suicidal ingestions was 47.5 mg (12.5–100 mg). The most common clinical effects were abdominal pain, oral irritation, throat irritation, nausea, dizziness, and headache. Nine patients received folinic acid and 3 patients received sodium bicarbonate. No patient developed renal failure, bone marrow suppression, seizure, or coma. No patient died or received dialysis. *Conclusion.* In our series of patients from 6 poison centers over six years, 63 cases of acute adult methotrexate ingestions were reported. Methotrexate toxicity from ingestion in adults was uncommon and rarely toxic.

## 1. Background

Methotrexate is a folic acid analogue and antagonist. The mechanism of action for methotrexate lies in its intrinsic ability to effectively block the synthesis of tetrahydrofolate from dihydrofolate through the inhibition of dihydrofolate reductase. Methotrexate utilizes several intercellular pathways to antagonize folate which results in profound effects on rapidly dividing cells [1].

Common uses of methotrexate include treatment for a variety of cancers. It is also used for rheumatoid arthritis, ankylosing spondylitis, uveitis, organ transplantation, psoriasis, trophoblastic diseases, and therapeutic abortion [1]. Methotrexate regimens for chemotherapy and therapeutic abortions are administered parenterally. Intrathecally, methotrexate is available in variable doses (1–20 mg/kg). For the treatment of rheumatological conditions including psoriasis, rheumatoid arthritis, and ankylosing spondylitis, the adult dose is 7.5–15 mg orally per week.

In past decades, isolated case reports of nonfatal toxic ingestion of methotrexate have been described. These cases did not have long term sequelae. More recently, oral methotrexate use has increased in frequency and dosage. In Germany, oral methotrexate has become established as the most commonly used disease modifying antirheumatic drug (DMARD) in the treatment of rheumatoid arthritis [2]. In the United States, a higher dose and early use of oral methotrexate for treatment of inflammatory arthropathy has been used [3]. Recent case reports describe fatal outcomes from acute methotrexate ingestion after repeated ingestions [4]. However, most cases of adverse effects are with parenteral administration or repeated oral ingestion. There is limited data on effects of acute ingestion of methotrexate in adults [5].

The objective of our study was to determine the frequency of significant toxicity effects after methotrexate ingestion in adults. In addition, we describe the frequency of methotrexate ingestions, treatments used, use of parenteral folate or folinic acid, hemodialysis, and systemic effects.

## 2. Materials and Methods

### 2.1. Study Design

We performed a retrospective cohort study of patients reported to a statewide network of poison centers with an exposure to methotrexate. The database of poison center records was searched using standard search criteria. Inclusion criteria were as follows: substance being methotrexate, adults aged 18 years and older, acute exposure, ingestion only, reported 01/01/2000–12/31/2005, and followed to a known outcome. All records were cleansed of any patient specific data. This study was approved by the Scott and White Healthcare institutional review board. Exclusion criteria were chronic exposures and doses delivered parenterally.

The Texas Poison Center network receives spontaneous phone calls from patients and health care providers. The network is composed of six poison centers that provide clinical consultative services throughout Texas 24 hours per day. Each call is received by trained nurses, physicians, or pharmacists for the purpose of managing the exposure and documenting the consultation. All cases are recorded in a nationally standardized data collection form maintained by the American Association of Poison Control Centers and stored in a database located at the Central Texas Poison Center. The database contains more than 180,000 per year. Cases are followed until the outcome of the event is known. Medical toxicologists are available for consultation on complex cases. If a patient is not referred to a health care facility, the poison specialist will call the patient back within one to three hours to obtain followup and determine resolution of symptoms. If the patient goes to the hospital, the specialist continues to follow the case by phone and collects medical information and course from the patient's provider until the patient is discharged or died.

Records of all methotrexate ingestions by patients over seventeen years old during 2000–2005 were collected from six poison centers. The cases consisted of all methotrexate ingestions, including those with coingestions. The poison center databases had recorded various clinical effects and interventions. One trained reviewer, blinded to the study purpose, used a standardized data collection form to extract study data. Missing or conflicting data were reconciled with a predetermined process.

### 2.2. Data Collection and Processing

Symptoms, signs, treatments, and outcomes were extracted from the database through chart review of the clinical notes and coded diagnoses and treatments. Outcomes were coded as “No effect”, “Minor”, “Moderate”, “Major”, and “Death” according to the American Association of Poison Centers National Poison Data System (NPDS) outcome criteria [6]. “No effect” is when the patient did not develop any signs or symptoms because of the exposure. “Minor effects” are signs or symptoms as a result of the exposure, but they were minimally bothersome and generally resolved rapidly with no residual disability or disfigurement. “Moderate effects” are signs or symptoms as a result of the exposure that were more pronounced, more prolonged, or more systemic in nature than minor symptoms. Usually, some form of treatment is indicated. Symptoms were not life-threatening, and the patient had no residual disability or disfigurement. “Major effects” are signs or symptoms as a result of the exposure that were life-threatening or resulted in significant residual disability or disfigurement (e.g., repeated seizures or status epilepticus, respiratory compromise requiring intubation, ventricular tachycardia with hypotension, cardiac or respiratory arrest, esophageal stricture, and disseminated intravascular coagulation). “Death” is when the patient died as a result of the exposure or as a direct complication of the exposure. Our network is made of six poison centers and calls are received and documented by trained poison information specialists. They enter clinical notes and document standardized codes for common symptoms, signs, and treatments. Free text clinical notes are also entered.

Records meeting the inclusion criteria were saved, printed, and reviewed. One trained abstractor abstracted each chart and entered the data into a secure spreadsheet. The abstractor reviewed the clinical notes for symptoms, signs, and treatments not coded. One of the investigators trained the abstractors and audited 30% of the charts. The abstractors trained on 10 charts were not included in the study cohort. They were given feedback on data abstraction and corrected mistakes. We devised the standard data collection tool before this run-in period and it was revised before the chart review began.

For a discrepant case on audit, two authors discussed the case after independent review of the record. Other data collection variables included demographics (age, gender), exposure reason, symptoms, signs, vital signs, treatments, systemic effects, symptom duration, and disposition. The NPDS defines tachycardia as a pulse rate greater than 100 beats per minute, and it defines hypertension as diastolic blood pressure greater than 90 mm Hg [7]. Definitions and outcomes were defined prior to abstraction. Agreement of abstraction was measured with the audited charts.

### 2.3. Outcomes Measures

Our primary outcome was incidence of methotrexate ingestion. Our secondary outcomes were frequency of renal insufficiency, abnormal liver tests, seizure, altered mental status, or use of specific treatments (intravenous bicarbonate, folinic acid, or hemodialysis). We also recorded other serious symptoms, signs, or treatments, such as hypotension, endotracheal intubation, or death.

### 2.4. Primary Data Analysis

All data was entered into a password-protected electronic spreadsheet (Microsoft Excel; Microsoft Corporation, Redmond, WA, 2007). We performed descriptive statistics.

## 3. Results

Sixty-three patients met our inclusion criteria of adult methotrexate ingestions. The mean estimated dose for all patients was 24 mg (median 12.5, range 2.5–100 mg). The mean patient age was 49 years (median 46, range 21–92 years). Eighty-eight percent (56/63) were acute ingestions. Seventeen percent (11/63) were male. Seventy percent (44/63) were solely methotrexate ingestions. Twenty-one percent (13/63) were intentional ingestions or suicide attempts. The mean estimate dose for intentional ingestions was 47.5 mg (median 52, 12.5–100 mg). Fifty percent (7/14) of the intentional ingestions received charcoal and four received gastric lavage. The most common symptoms for all patients were mild and included abdominal pain, throat irritation, oral irritation, nausea, and headache (Table 1). Most had no symptoms (43/63, 68%).

No patients developed seizure, lethargy, vomiting, dysrhythmia, or renal failure. Two patients had abnormal liver enzymes; however, one had a history of chronic liver disease and the other patient ingested acetaminophen and presented more than 8 hours after ingestion. Nine patients (9/63, 14%) received folinic acid and 3 of those patients (3/63, 5%) also received intravenous sodium bicarbonate (Table 2). No patient in the group receiving folinic acid had serious adverse effects attributable to methotrexate toxicity. Hemodialysis or deaths were not reported in the record. No renal insufficiency, bone marrow suppression, or seizures were reported either. Followup more than 12 hours after ingestion occurred in 19% of cases. Kappa statistic was performed for interrater reliability agreement and was 0.80 for primary outcome, use of therapy, and clinical effect. Most patients have no effects or minor/minimal effects (Table 3).

*Limitations. *Our study is a retrospective database review and contains the inherent limitations. However, we attempted to mitigate the retrospective study design flaws with strict methodology using two trained abstractors for each chart, holding periodic meetings, using a standardized data form, and measuring interrater agreement. Exposure records housed in the Texas Poison Center network database are from self-reported calls. Information contained in the record reflects only the information shared by the public caller or healthcare provider and may not be complete. Records in this database do not necessarily represent a poisoning or overdose; rather they represent a reported exposure. Our study is also limited by the use of Texas Poison Center network records and strict categories for clinical effects. Only patients and providers who called the poison center were evaluated possibly limiting the spectrum of cases reviewed. Patients may have visited a health care facility and not called a poison center. Some of these cases may have had renal or bone injury or required hemodialysis. Hospital and clinic medical records were not available for review, limiting conclusions on systemic findings or therapies given. In addition, poison control centers do not maintain records of certain exam findings, abnormal vital signs, and the fact that treatments were not missed. Like other studies, we used the clinical effect categories listed by the poison control center as well as the clinical notes to determine outcomes and clinical course. Each patient care notes was reviewed for treatments or clinical effects. Many patients had no follow-up after the initial consultation with the poison control center. Some of these patients may have had an adverse outcome. Methotrexate levels were not available for review. Finally, follow-up less than one week in most cases may have prevented knowledge of delayed effects of methotrexate toxicity.

## 4. Discussion

The results of our study show that methotrexate toxicity is an uncommon occurrence. In our 6-year review of methotrexate ingestions, we report data from 63 patients, demonstrating that methotrexate ingestions are uncommon. Furthermore, our series revealed non-life-threatening signs and symptoms such as abdominal pain, oral irritation, throat irritation, nausea, dizziness/vertigo, and headache which have been described. None of the patients we describe demonstrated the more severe sequelae such as renal failure, sepsis, or respiratory failure that have been reported as the result of intravenous and repeated oral exposures.

Methotrexate has been used in parenteral form as a chemotherapeutic agent for decades. In recent years, the oral form has been used for the treatment of rheumatologic conditions with both increased frequency and dosage [8]. Parenteral methotrexate toxicity has been examined, and several effects such as renal failure, respiratory failure, myelosuppression, and neurologic disruption have been described [1, 9]. Recent case reports have described fatal overdose of methotrexate ingestions, although no data from a large series of acute ingestions has been reviewed until now [4].

While several antidotes have been postulated for methotrexate toxicity, folinic acid (leucovorin) has been shown to be the most effective and has few known complications [10]. We found that the use of methotrexate-specific treatments was uncommon, possibly suggesting that physicians were unfamiliar with this rarely used drug or the physicians were not concerned the patients were ill and need it.

In this series, we found that 21% of cases resulted from suicidal ingestions. Although this subgroup ingested twice as much methotrexate (24 mg versus 47.5 mg), no unique clinical effects were observed. Yet given the potentially severe effects of methotrexate and its increasing use in oral form, further study is warranted to elucidate its adverse effects.

LoVecchio et al. reported 13 adults and children who had methotrexate exposure [5]. Our study had similar results to LoVecchio, in the fact that no patients had severe effects. However, our study examined only adults and includes four times more patients than their adult group, making our results more convincing that acute oral ingestion of methotrexate does not cause serious effects. In addition, in our study, the mean dose ingested was higher (24 mg for all subjects and 47.5 mg for intentional ingestions). We also have a larger proportion of suicidal patients in study (22% versus 15%). Finally, our annual volume of poison center calls is greater (180,000 cases per year versus 50,000 cases) allowing for a large volume of cases of methotrexate to be evaluated.

Based on our study and LoVecchio's case series, the incidence of methotrexate toxicity from acute ingestion in adults is uncommon. In addition, the incidence of adverse effects is also rare and most patients develop mild symptoms. Based on these findings, supportive care and observational therapy without methotrexate-specific treatment should be considered in acute ingestions. Patients can be monitored for development of renal insufficiency, neurologic effects, or bone marrow suppression. Based on the clinical effects and followup, the measurements and reexaminations could be done as an inpatient or outpatient. A large, prospective study to evaluate this treatment approach is warranted.

In conclusion, in our largest study of acute methotrexate ingestions, reported cases of acute methotrexate ingestion are uncommon. In addition, serious toxicity was not reported and use of methotrexate-specific treatment was rare after acute methotrexate ingestions. Observation without methotrexate-specific treatments should be considered in acute methotrexate ingestions.

## Figures and Tables

**Table 1:** Clinical effects of acute methotrexate ingestions in adults as reported to six poison centers (*N* = 44).

Clinical effects	Number of patients	Percentage of patients
Any clinical effect	13	29.5%
Chest Pain	1	2.2
Tachycardia	1	2.2
Abdominal Pain	2	3.2
Rash	1	2.3
Diarrhea	1	2.3
Throat irritation	3	6.8
Oral irritation	2	3.2
Nausea	2	3.2
Dizziness/vertigo	2	3.2
Headache	2	3.2
Lethargy	0	0
Seizures	0	0
Renal insufficiency	0	0

**Table 2:** Therapies reported for acute methotrexate ingestions in adults as reported to six poison centers (*N* = 44).

Therapy	Number of patients	Percentage of patients
Any treatment	24	54%
No treatment	20	46
Dilute irrigation/wash	14	22.2
IV fluids	11	18
Cathartic	10	16
Charcoal-one dose	9	14.3
Folinic acid	9	14.3
Lavage	5	8
Charcoal-multiple doses	3	4.8
Sodium bicarbonate	3	4.8
N-acetyl cysteine (oral)	3	4.8
Antiemetics	2	3.2
Hemodialysis	0	0

**Table 3:** Outcomes reported for acute methotrexate ingestions in adults as reported to six poison centers (*N* = 42 with outcomes reported out of 44 cases).

Outcome	Number of cases (%)
No effects	9 (21%)
Minor or minimal effects	30 (71%)
Moderate effects	1 (2%)
Major effects	1 (2%)
Death	0
